# Self-care among caregivers of people living with HIV and AIDS in Kakola location, Nyando District, Kisumu County, Kenya

**DOI:** 10.1080/17290376.2013.807065

**Published:** 2013-10-04

**Authors:** Leila Moraa Geteri, Evelyn Mandela Angogo

**Keywords:** self-care, primary caregivers, Kenya, PLWHAs, HIV and AIDS, Soin de soi parmi, persennol soignant primaire, Kenya, PLWHAs, Le VIH et le SIDA

## Abstract

This study was carried out in Kakola Location of Nyando District in Kenya. The aim of study was to determine the factors influencing the practice of self-care among caregivers for person living with HIV/AIDS (PLWHAs) as well as their practice of self-care. A study by World Health Organization approximated that in developing countries, the need for long-term care will increase by as much as 40% in the coming years. HIV/AIDS has been cited as one of the challenges in long-term care. As demand for long-term care increases, the assumption that extended family networks can meet all the needs of their members deteriorates. The community-based survey employed descriptive cross-sectional design, involving primary caregivers of PLWHAs in Kakola location who had practiced care giving for more than 3 months. A household survey was conducted with 150 respondents. Quantitative data were analyzed using the Statistical Package for Social Sciences (SPSS) program version 11.0. Simple frequencies and cross tabulations to compare variables were produced. Microsoft Excel was used to produce tables and graphs. Majority of the respondents 124 (82.7%) were female, while 26 (17.3%) were male. Self-care elements most practiced by the respondents in all the age categories were infection prevention and nutritional care. Female respondents had the highest proportions in all the practices of self-care. The results also showed that gender, relationship of patient to caregiver and marital status were the main demographic factors that significantly influenced the practice of self-care among caregivers. There was a significant relationship between main sources of income of caregivers with the practice of self-care. The study also revealed that respondents with no education had the lowest number of respondents practicing all the six practices of self-care and belonging to a support group. Recommendations for the study included, forging partnerships among stakeholders, training of caregivers and review of the home-based care policy.

## Abstract (FR)

Cette étude a été effectuée dans l'emplacement Kakola du district Nyando au Kenya. Le but d'étude était de déterminer les facteurs influençant la pratique de soin de soi parmi le personnel soignant pour PLWHAs aussi bien que leur pratique eux-mêmes. Une étude par OMS, montre que dans des pays en voie de développement, le besoin du soin à long terme augmentera de bien 40% dans les années à venir. Le VIH/SIDA a été cité comme un des défis dans le soin à long terme. Comme exigent les augmentations de soin à long terme, la supposition que les réseaux de famille étendue peuvent se réunir tous les besoins de leurs membres se détériore. L'enquête à base de communauté a employé la conception à éléments mutuel descriptif, impliquant le personnel soignant principal de PLWHAs dans l'emplacement Kakola qui avait pratiqué le don de soin pendant plus de 3 mois. Une enquête du ménage a été conduite avec 150 défendeurs. Des données quantitatives ont été analysées utilisant le Paquet Statistique pour la version 11.0 de programme de Sciences Sociales. Des fréquences simples et des tabulations mutuelles pour comparer des variables ont été produits. Microsoft Excel a été utilisé pour produire des tables et des graphiques. La Majorité des défendeurs 124 (82.7%) était féminine tandis que 26 (17.3%) étaient masculins. Les éléments de soin de soi le plus pratiqué par les défendeurs dans toutes les catégories d'âge étaient la prévention d'infection et le soin nutritionnel. Les défendeurs féminins avaient les dimensions les plus hautes dans toutes les pratiques de soin de soi. Les résultats ont aussi montré que le genre, la relation des malades à la personne donnant des soins et la situation de famille étaient les facteurs démographiques principaux qui ont beaucoup influencé la pratique de soin de soi parmi le personnel soignant. Il y avait une relation majeure entre les sources principales de revenu de personnel soignant avec la pratique de soin de soi-même. L’étude a aussi montré que les défendeurs sans éducation avaient un effectif le plus bas des défendeurs pratiquant toutes les six pratiques de soin de soi et appartenant à un groupe d'entraide. Recommandations pour l’étude incluse, établir des partenariats parmi parties prenantes, recevant une formation de donneurs de soin et examiner la politique d' soins à domicile.

## Introduction

Long-term care for people with chronic illnesses and disabilities is a pressing issue globally. A study by World Health Organization (WHO) approximated that in developing countries, the need for long-term care will increase by as much as 40% in the coming years (WHO 2002). This issue has been heightened by a combination of demographic and epidemiologic forces. HIV/AIDS has been cited as one of the challenges in long-term care. The existing systems of informal long-term care have come under great strain, some have already begun to untangle. Who, then, will carry the burden? Existing systems of care at the community level, which typically depend on unpaid family members, are not enough to meet the growing demand for care. Although families continue to play an integral role in providing long-term home-based care, countries must ensure that resources are available to address the growing need for long-term care (WHO 2002)

A caregiver in the HIV and AIDS context is anyone (professional, lay or family) involved in taking care of the physical, psychological, emotional and/or spiritual needs of a person living with HIV/AIDS (PLWHAs) (Fekadu, Jari & Merja 2009; Van Dyke 1999). In the formal health sector, caregivers are usually nurses, counselors and social workers, but Africa has so many PLWHAs that hospitalization or formal care is strained. The enormous need for care leaves the community with no choice but to care for some of its own sick members (Fekadu *et al.* 2009). Care giving burden is a vital concern and will become more so with the inevitable aging of the population. Caregivers must be able to recognize those factors associated with caregiver burden to give quality care to their patients and families (Donelan, Hill, Hoffman, Scoles, Feldman, Levine *et al.* 2002).

It was estimated that by the end of 2007, there were 33.2 million PLWHAs of which, 30.8 million were adults, 15.4 million were women, and 2.5 million were children. Sub-Saharan Africa has just over 10% of the world's population, but is home to more than 61% (22.5 million) of all people living with HIV. In 2007, an estimated 1.7 million people in the region became newly infected, while 1.6 million adults and children died of AIDS-related conditions (UNAIDS 2007).

Sometimes, older children help to care for their sick parents and to raise their siblings in addition to performing daily chores like fetching water and herding animals. They cook, take care of their siblings, do the washing and most importantly, nurse their sick parent. With this extra responsibility, there is little or no time left for school work or play leading to absenteeism and sooner to school dropout. The added responsibility may overwhelm them and by providing nursing to their sick parents without having been taught what to do, they also risk being infected because they do not know what precautions to take (Mallmann 2003).

There has been an increase in the number of third-generation caregivers. A third-generation caregiver refers to grandparents caring for their sick daughters and their grandchildren. A study carried out in Budalangi in Busia district on 40 households revealed that the number of third-generation caregivers had risen sharply within the last two years preceding the study. The findings further revealed that majority of the grandparents were nursing an ailing son or daughter prior to their death (Mutere 2003). A study identifying stressors felt by volunteers for PLWHAs, and determining the types of social support needed to aid volunteers cope with the stressors, was done in Appalachia with six volunteers in a small non-profit organization providing such services. The results indicated that even though care giving is a rewarding experience, caregivers of PLWHAs often experience stress and frustration and social support may help in alleviating this stress (Held & Brann 2007). Another study was conducted on women who were family caregivers of spouses with AIDS, living in the Bumbu Zone, Kinshasa, and Democratic Republic of Congo. Eighty caregivers were randomly selected from a client visitation list of the home-based care (HBC) program for AIDS patients, and interviewed using a semi-structured questionnaire. The results showed that self-reported poor health was higher in female caregivers. Poor health was linked to low income, rented accommodation, little support, and stigmatization of the caregiver by relatives (Nkosi & Kipp 2008). In an exploratory and descriptive study, which explored the experiences and effect on young girls and older women caring for family members living with HIV and AIDS and other chronic and terminal illnesses at home in three districts of Botswana; using qualitative research methods, 70 interviews were conducted with family caregivers and key informants such as community HBC team members and government officials. The results of the study showed that older women experienced exhaustion, depression and often neglected their own health, while in the young girls' school absenteeism, stigma and stress were noted. Both experienced stigma, poverty, and a lack of basic care giving teaching (Lindsey, Hirschfeld & Tlou 2003).

Caring for another person takes a lot of time, effort, and work, and most caregivers alternate care giving with full-time jobs and parenting and in the process, caregivers put their own needs aside (Macmillan, Peden, Hopkinson & Hycha 2004).

## Statement of the problem

Caregivers are vital in the care of PLWHAs due to the overwhelming and increasing number of PLWHAs; therefore, there is an increasing need for care. The burden of care in addition to other responsibilities that the caregiver has, has a profound effect on the health of the caregiver. Care giving in itself makes them more vulnerable to physical, emotional and psychological ailments and therefore there is a critical need for them to be able to cope with the care giving stress to prevent burnout and exposure to infection.

The need for coping mechanisms for caregivers led to the development of national HBC guidelines in the year 2002 and in these guidelines, there is a section that deals with the care of caregivers. This care includes prevention of infection, counseling, and respite. Whether the caregivers properly adhere to these guidelines has not been properly documented; the guide also does not provide for nutritional care, exercise, and care-seeking practices and therefore, the need for this study. The study was, therefore, designed to explore not only knowledge of caregivers, but also their practice of self-care.

In the study, self-care is used to describe a process in which people actively function on their own behalf in health promotion and prevention, and in disease detection and treatment. Self-care practices included elements such as prevention of infection; nutritional care; respite; exercising; care seeking; and psychological care.

In view of the picture portrayed above, providing answers to the following questions can fill the knowledge gap and facilitate improved care of caregivers.

(1) What demographic, socio-economic, socio-cultural, health service factors influence the practice of self-care among caregivers of PLWHAs?
(2) What is the knowledge and practice of self-care among caregivers of PLWHAs?

## Objective of the study

The objective of this study was to determine the factors influencing the practice of self-care among caregivers of PLWHAs as well as their practice of self-care in Kakola Location of Nyando District.

## Methodology

This was a descriptive study that utilized quantitative methods to establish factors influencing the practice of self-care among caregivers of PLWHAs.

### Population

The study population was drawn from self-identified primary caregivers of PLWHA in Kakola location, Nyando district. The criteria for inclusion were that: primary caregivers had cared for PLWHA for more than 3 months in the area of study.

### Sample and sampling

The sampling design was convenient for all respondents using snowballing. This technique was chosen due to the stigma attached to HIV and AIDS. Community health workers (CHWs) working with PLWHAs in Kakola sub-location helped identify some of the caregivers, who in turn helped enumerators to identify other caregivers. The study utilized a complete sample of 150 primary caregivers who agreed to participate in the study.

## Ethical consideration

Before the commencement of data collection, clearance to carry out the research was obtained from the ethics and research committee at the Great Lakes University of Kisumu, Ministry of Education, Ministry of Health, and other relevant authorities.

Informed verbal consent was obtained from participating caregivers with the assurance of confidentiality during and after the study. Content of the informant consent form included: general introduction of the study, usefulness of the study, and purpose of the study. Codes were used instead of names in order to protect the identity of respondents.

## Quality control

The study instrument was checked and approved by the assigned supervisors. Translation of tools to local vernacular was done to suit the respondents (Dholuo). Before commencement of data collection, the enumerators and research assistant were standardized though training on how to use the research instruments and how to record data to reduce bias and errors during data collection. All data were cleaned and edited at the end of each day. Pilot testing was done after training to ensure validity and reliability of the information to be collected.

## Data collection

Quantitative data collection took 4 days. Data were collected using structured questionnaires, which were administered to the 150 primary caregivers. The structured questions were built on demographic, economic, and socio cultural.

Considering the stigma surrounding PLWHAs and legal issues, enumerators were selected from outside the community, this was to enable gathering of information to be done as freely as possible without the respondents worrying about other community members knowing what they had discussed. Enumerators were selected based on their past experience in community work, health background, or work relating to HIV and AIDS, fluency in Dholuo, Kiswahili, and English. A total of 16 enumerators were chosen and trained for 2 days on what the study was about, its purpose, the study procedures and the instrument to be used. Guides were selected from the community through their key persons.

Enumerators liaised with the CHWs from Kakola sub-location who introduced them to the first caregiver who in turn led them to the other caregivers.

## Data management and data analysis

SPSS was used to analyze the data. The screen was set and measures of scale were assigned for all variables before data entry. Data were entered. Post-data entry cleaning was done by identifying wrong entries and correction was done with reference to questionnaires. This was to ensure that the data were accurately entered to avoid any errors during data analysis.

Data processing was done in three steps, data cleaning, post coding, and data analysis. Data analysis was done using SPSS and Microsoft Excel. Descriptive methods of statistical analysis were used, which included: frequencies and percentages. The findings were presented in the form of cross-tabulations, tables, and graphs. Significant statistical tests were carried out using Pearson chi-square.

## Findings and discussion

### Demographic factors influencing practice self-care

Demographic information included: age and sex of the respondents, relationship of caregiver to the client, number of dependents and marital status, *n* = 150.

Respondents were of different age groups. Thirty-seven percent of the respondents were aged between 16 and 30; 32% of respondents were in the age range between 31 and 45 years; 26% were between the ages of 46 and 60, and 5% were between 61 and 65 years. Caregivers aged between 16 and 45 years were more likely to practice all six self-care practices than caregivers aged between 61 and 75 and underage caregivers. These findings are consistent with a study by Lindsey *et al.* (2003) in Botswana, showing that older women experienced exhaustion, depression, and often neglected their own health, while in the school absenteeism, stigma and stress were noted, among young girls.

Self-care elements most practiced by the respondents in all the age categories were the prevention of infection and nutritional care (Table 1).

**Table 1.** Practice of the self-care by number of dependents.

	Practice of self-care (% within practice of self-care)					
Number of dependents	Prevention of infection, *n* (%)	Nutritional care, *n* (%)	Respite, *n* (%)	Exercise, *n* (%)	Care seeking, *n* (%)	Psychological support, *n* (%)
1 (*n* = 1)	1 (0.9)	1 (0.8)	0 (0)	1 (2.0)	0 (0)	0 (0)
2–3 (*n* = 19)	15 (13.0)	17 (12.9)	4 (18.2)	10 (20.0)	6 (13.3)	6 (13.3)
4–5 (*n* = 47)	36 (31.3)	42 (31.8)	5 (22.7)	15 (30.0)	18 (40.0)	19 (42.2)
>6 (*n* = 83)	63 (54.8)	72 (54.5)	13 (59.1)	24 (48.0)	21 (46.7)	20 (44.4)
Total (*n* = 150)	115 (100)	132 (100)	22 (100)	50 (100)	45 (100)	45 (100)

#### Sex of caregiver

Out of 150 respondents 26 (17.3%) were males and 124 (82.7%) were females. Male respondents who practiced prevention of infection were 69.2%, while female respondents who practiced prevention of infection were 78.2%. In exception of practice of respite care, where the proportion of male respondents practicing it was higher (19.2%) than female respondents (13.7%), female respondents had a higher proportion in all the other practices.

More than half of the respondents (*n* = 150), both male (57.7%) and female (66.1%) had more than two meals per day; there was no statistical significant relationship between gender and the number of meals taken per day; the *p*-value generated was 0.077. In comparing gender and specific prevention of infection practices, hand washing and gloving were practiced by majority of the respondents. Female respondents accounted for a higher proportion (79.6%) in the practice of hand washing, while male respondents accounted for a higher proportion (76.9%) in the practice of gloving. Gender was statistically significant in the practices of hand washing, where the *p*-value was 0.005 and also in the practice of gloving where the *p*-value was 0.018; whereas, it was not significant in the practice of proper waste disposal (*p* = 0.284). The study established that the main caregivers were women of reproductive age and that women were more likely to take up care giving roles than males, as there were more women respondents. Findings, which are consistent with VSO (2006), which stated that women, children and grandparents have taken or been forced to take the role of caregiver in their communities, it goes on to say that the women's productive capacity is decreased or lost when they become caregivers. This study also established that women were more likely to practice all the self-care practices.

### Marital status of caregiver

Almost half where *n* = 150, 48.7% of respondents were married, 34.7% were widowed, 14.7% were single, and 1.3% were separated with their spouses, while 0.7% were under 15 years. Respondents who were married had the highest proportion (50.4%) in the practice of prevention of infection, 47.7% in the practice of nutritional care, 50% in the practice of respite, and 55.6% in the practice of care seeking. The relationship between marital status and exercising was found to be statistically significant as *p* = 0.029. The findings were consistent with a study done by Nkosi, Kipp, Laing and Mill (2006), where female caregivers giving care to their spouses reported to having poor health.

### Relationship of caregiver to client

Most of the respondents were giving care to their relatives, 18.0% of the respondents were giving care to their spouses, 3.3% to their fathers, 18.7% to their mothers, 18.0% to their sons, 18.0% to their daughters, and 10.7% of the respondents were giving care to other relatives (*n* = 150). Those respondents caring for their fathers, mothers, and brothers did not practice respite. Those respondents caring for sons had the highest proportion in the practice of prevention of infection (21.7%), respite (27.3%), exercise (28%), and psychological support (31.1%). The relationship to patient was a statistically significant factor in the practice of hand washing where the *p*-value generated was 0.034, while there was no statistically significant difference in the practices of proper waste disposal where the *p*-value was 0.270 and gloving where the *p*-value was 0.213. Family relationship was noted to play a big role in the practice of care giving. Majority of the respondents were giving care to their nuclear family members. The relationship to client was a significant factor in the practice of prevention of infection. These findings agree with those of a study conducted by Gwoswar (2002) in Kisumu district. The study showed that half of caregivers were family members.

### Number of dependants

One (0.7%) respondent reported having 1 dependant, those having 2–3 dependants were 19 (12.7%), while those who reported having 4–5 dependants were 47 (31.3%) and those with more than 6 dependants were 83 and accounted for 55.3% of the total number of respondents (*n* = 150).

Respondents with more than six dependants accounted for the highest proportion in all the practices of self-care, while those with one dependant had the lowest proportion in all the practices of self-care. Majority of the respondents had more than six dependants (see Table 1). In this study, those respondents with more than six dependants had the highest proportions in all the six practices of self-care. Findings which were contrary to a study by Vithayachockikhun (2006) observed that as care demand increases, the care burden increases.

### Socio economic factors influencing the practice of self-care

Socio economic factors invested included: education and main source of income.

#### Education level of caregiver

Primary education accounted for the highest proportion (60.7%), followed by secondary education (23.3%), while 12.7% had no education and tertiary education accounted for 3.3% (*n* = 150).

Respondents with tertiary and those with no education had the lowest proportions in all the six practices in self-care. Education was statistically significant in the practice of respite (*p* = 0.028). Those with primary education had the highest proportion in all practices of self-care.

The results further showed that in the practice of prevention of infection, those with primary education had the highest proportion in all the specific practices of prevention of infection. Education was statistically significant in the practice of gloving, the *p*-value generated was 0.012, while there was no statistical difference in the practices of hand washing (*p* = 0.144) and waste disposal (*p* = 0.051) (see Table 2).

**Table 2.** Practice of the self-care by the level of education of the caregiver.

	Practice of self-care (% within practice of self-care)					
Level of education of the caregiver	Prevention of infection, *n* (%)	Nutritional care, *n* (%)	Respite, *n* (%)	Exercise, *n* (%)	Care seeking, *n* (%)	Psychological support, *n* (%)
None (*n* = 19)	11 (9.6)	15 (11.4)	2 (9.1)	3 (6.0)	4 (8.9)	3 (6.7)
Primary (*n* = 91)	74 (64.3)	81 (61.4)	11 (50.0)	28 (56.0)	31 (68.9)	31 (68.9)
Secondary (*n* = 35)	27 (23.5)	32 (24.2)	6 (27.3)	17 (34.0)	8 (17.8)	11 (24.4)
Tertiary (*n* = 5)	3 (2.6)	4 (3.0)	3 (13.6)	2 (4.0)	2 (4.4)	0 (0)
Total (*n* = 150)	115 (100)	132 (100)	22 (100)	50 (100)	45 (100)	45 (100)

#### Main source of income of caregiver

Majority of the caregivers' main source of income came from farming, which accounted for 36.7%, business and casual labor accounted for 27.3% each, then salary and none with 4% each and others which accounted for 0.7%. Respondents whose main source of income was farming had the highest proportion in the practices of prevention of infection (34.8%), nutritional care (39.4%), care seeking (57.8%), and psychological care (42.2%). Farming also accounted for 61.8% of those who had more than two meals/day, while 38.2% had two meals/day. Less than a quarter (14.3%) of respondents with no main source of income had one meal/day, 42.9% had two meals/day and 42.9% had more than two meals a day, the *p*-value generated was 0.003. Main source of income was found to be a statistically significant factor in the practice of care seeking.

### Socio-cultural factors influencing the practice of self-care

Social cultural factors investigated were: religion, membership in a support group.

#### Religion of the caregiver

Majority of the respondents (71.3%) were protestant, 28.7% were catholic. Protestants had the highest proportions in all the practices of self-care. Practices in the Catholics had a higher proportion in the practice of respite (16.3%) and care seeking (16.3%). The relationship between religion and psychological support was found to be statistically significant, *p* = 0.020.

#### Support group membership of the caregiver

Three quarters of the respondents(75%) belonged to a support group; 11.3% of the respondents belonged to a HBC support group, 31.3% belonged to a church group, 23.3% belonged to either a women/men's group, 6.7% to a youth group, while 27.3% did not belong to any support group (*n* = 150).

Respondents who belonged to church support groups had the highest proportion in four of the practices of self-care; practice of nutritional care (29.5), practice of prevention of infection (33%), respite (45.5%), and exercising (34%), while those who belonged to either a women/men group had the highest proportions in two of the practices of self-care; care seeking (46.7%) and psychological care (35.65). Support group membership was a statistically significant factor in the practices of care seeking (*p* = 000), and psychological support (*p* = 0.031). The type of support received by caregivers played a significant role in the practice of prevention of infection. Slightly above, 30% of the caregivers belonged to a church group, while 72.7% of caregivers belonged to a support group, showing the willingness of and the need to affiliate, share and partner with other people. A study done by Held and Brann (2007), in Appalachia, identified stressors felt by volunteers for PLWHAs and determined the types of social support needed to aid volunteers cope with stressors showed that even though care giving is a rewarding experience to caregivers of PLWHAs they often experienced stress and frustration and that social support could alleviate the stress felt by caregivers.

### Knowledge of caregiver

Knowledge of caregiver was assessed in six areas, prevention of infection, nutrition, exercising, respite, care seeking, and psychological support.

The respondents were asked if they knew what the concept of ‘care of caregivers’ means and 80.7% reported having knowledge of care of caregivers. The findings also showed that respondents who were aged between 16–30 and 31–45 years had the highest proportion (35.5% respectively) on knowledge of the care of caregivers, while those aged between 0 and 15 years had the lowest proportion (0.8%); however, age was not a statistically significant factor in the knowledge of care of caregivers. The results also showed that majority (81%) of the females had knowledge about the care of caregivers, while only 19% of the males had knowledge on the care of caregivers.

Respondents who had knowledge on the prevention of infection had the highest proportions in all the specific practices of prevention of infection. Knowledge was a statistically significant factor in the practice of gloving in the prevention of infection, *p* = 0.028. The study showed that education played an important role in the practice of self-care and respondents with primary education were more likely to practice all six practices of self-care. Education was seen to play a significant role in the practice of prevention of infection especially in reference to proper waste disposal, the practice of gloving when handling secretions, wounds and blood and also in the practice of respite. These findings agree with those of Kalra, Evans, Perez, Melbourne, Patel, Knapp, *et al.* (2004) stating that training decreases caregivers' burden, anxiety and showed an increased quality of life. Most of the caregivers reported having knowledge of care of the caregiver. The findings also demonstrated that respondents who were aged between 16 and 45 years had the highest proportion on knowledge of the care of caregivers while those aged between 0–15 years and 61–75 years had the lowest proportions. These findings agree with those of Juma, Okeyo & Kigenda (2004) that many older adults lack essential knowledge, skills, and resources for patient care and that many elderly caregivers care for the sick with limited knowledge of HIV/AIDS which may affect their own health and that of their patients

### The practice of self-care

#### Practice of prevention of infection

Almost three quarters of the respondents (76.7%) practiced prevention of infection (*n* = 150). In the practice of prevention of infection, 75.3% of the respondents practiced hand washing, 48.0% practiced proper waste disposal, and 56.0% practiced gloving.

#### Respite

Majority of the respondents (64.0%) had one patient, 18.7% had two patients, and 17.3% had more than two patients. Most of the respondents (81.3%) took time off patient care to rest. Among those who took time off from care giving to rest, 63.1% had one patient, 18.9% had two patients, and 18.0% had more than two patients. Majority of those who took time off had one patient followed by those with two patients and then those with more than two patients implying that the more patients one has the less likely they are to take time off. When asked if they had put off seeking care because of care giving activities, 22.7% of the respondents said they did. This finding agrees with a study by Macmillan *et al.* (2004), showing that caring for another person takes a lot of time, effort and work, plus most caregivers alternate care giving with full time jobs and parenting, in the process, caregivers put their own needs aside.

#### Care seeking

Majority of the respondents (60.7%) reported having been sick in the past 4 weeks, while 39.3% well (*n* = 150). When asked if they had put off seeking health care because of care giving activities, 22.7% of the respondents said they did, while 77.3% did not.

## Conclusion

Study results have shown that, gender of the caregiver, familial relationship of caregiver to PLWHA, and marital status were the main demographic factors that significantly influenced the practice of self-care among caregivers. The number of dependants a caregiver had played a major role in the dedication of the time a caregiver had to practice self-care as caregivers had dual roles caring for their patient and caring for their families.

Education was found to be very significant in all the practices of self-care. The occupation and main source of income of caregivers influenced the type of practice they engaged in. Caregivers who had businesses practiced prevention of infection and nutritional care more than the other practices; farmers practiced nutritional care more than the others. Religion plays an important role in the lives of caregivers as all of the caregivers were Christians by religion, all of who got spiritual support from their various churches.

Belonging to a support group also influenced the practice of self-care especially in the practice of respite and psychological care. Majority of the caregivers reported having knowledge of the care of caregivers. Though majority of the respondents had knowledge of specific practise of prevention of infection and practiced it, knowledge was only statistically significant in the practice of gloving.

Finally, but not least prevention of infection and practice of nutritional care are the most practiced self-care practices.

### Recommendations

The health facility/hospital and NGOs should forge a partnership with the caregivers and PLWHAs in order to assess, plan, and act on issues affecting this population. There is a need to train caregivers on the importance of the care of the caregiver, therefore, programmes need be set up to sensitize and educate caregivers on the importance of respite, exercise, care seeking, and psychological support/counselling in decreasing and reducing stress and burnout. The number of patients under the care of a caregiver should not be more than two.

The Governments should review the HBC policy so as to include a comprehensive and in-depth look at the care of caregivers and also come up with strategies that will increase the practice of care of caregivers in all areas, namely at the facility level and household level. Ministry of Health should strengthen the community participation in such a way as to develop linkages between caregivers and the health facilities. The role of primary caregivers should be recognized as an integral part in the care of caregivers. Finally, further research should be done to determine factors influencing health status of caregivers.

## References

[R1] Donelan K., Hill C.A., Hoffman C., Scoles K., Feldman P.H., Levine C. (2002). Challenged to care: informal caregivers in a changing health system. Health Affairs.

[R2] Fekadu A., Jari K., Merja N. (2009). The conceptions of care among family caregivers of persons living with HIV/AIDs in Addis Ababa, Ethiopia. Journal of Transcultural Nursing.

[R3] Gwoswar A.C. (2002). Linkage in Continuum of Care of People Living with HIV/Aids: A Case Study of Kisumu Municipality.

[R5] Held M.B., Brann M. (2007). Recognizing HIV/Aids volunteer stressors and desire for support. Aids Care.

[R6] Juma M., Okeyo T., Kigenda G. (2004). Our Hearts Are Willing, but… Challenges of Elderly Caregivers in Rural Kenya. Horizons Research Update.

[R7] Kalra L., Evans E., Perez I., Melbourne A., Patel A., Knapp M. (2004). Training carers of stroke patients: randomized controlled trail. BMJ.

[R8] Lindsey E., Hirschfeld M., Tlou S. (2003). Homebased care in Botswana: older women and young girls. Health Care for Women International.

[R9] Macmillan K., Peden J., Hopkinson J., Hycha D. (2004). A Caregiver's Guide: A Handbook about End of Life Care.

[R10] Mallmann A. S. (2003). Catholic AIDS9 Action. Building Resilience in Children Affected by HIV and AIDS.

[R11] Mutere B. (2003). The Effects of AIDS: Induced Orphanhood on Third Generation (grandparent), Caregivers: A Case Study of Budalangi Sub-location, Busia District.

[R12] Nkosi T. M., Kipp W. (2008). Factors Associated with the Self-Reported Health Status of Female Caregivers of Aids Patients. Western Journal of Nursing.

[R13] Nkosi M. T., Kipp W., Laing L., Mill J. (2006). Family Care Giving for Aids Patients in the Democratic Republic of Congo.

[R14] UNAIDS (2007). Report on the Global Aids Epidemic.

[R15] Van Dyke V.A. (1999). HIV and AIDS Care and Counseling. A Multidisciplinary Approach.

[R16] Vithayachockikhun N. (2006). Family care giving of people living with HIV/Aids in Thailand: caregiver burden an outcome measure. International Journal of Nursing Practice.

[R17] VSO (2006). Reducing the Burden of HIV/Aids on Women and Girls. VSO policy brief.

[R18] World Health Organization (2002). Ethical Choices in Long-term Care: What does Justice Require?.

